# Study on Single Event Effects of Enhanced GaN HEMT Devices under Various Conditions

**DOI:** 10.3390/mi15080950

**Published:** 2024-07-24

**Authors:** Xinxiang Zhang, Yanrong Cao, Chuan Chen, Linshan Wu, Zhiheng Wang, Shuo Su, Weiwei Zhang, Ling Lv, Xuefeng Zheng, Wenchao Tian, Xiaohua Ma, Yue Hao

**Affiliations:** 1 School of Electronics & Mechanical Engineering, Xidian University, Xi’an 710071, China; 22041212821@stu.xidian.edu.cn (X.Z.); 22041212707@stu.xidian.edu.cn (C.C.); 22043222985@stu.xidian.edu.cn (L.W.); 21041211891@stu.xidian.edu.cn (Z.W.); 23041212658@stu.xidian.edu.cn (S.S.); 23041212555@stu.xidian.edu.cn (W.Z.); wctian@xidian.edu.cn (W.T.); 2 State Key Discipline Laboratory of Wide Bandgap Semiconductor Technology, School of Microelectronics, Xidian University, Xi’an 710071, China; llv@xidian.edu.cn (L.L.); xfzheng@mail.xidian.edu.cn (X.Z.); xhma@xidian.edu.cn (X.M.); yhao@xidian.edu.cn (Y.H.)

**Keywords:** GaN HEMTs, single event transient effects, charge collection

## Abstract

GaN HEMT devices are sensitive to the single event effect (SEE) caused by heavy ions, and their reliability affects the safe use of space equipment. In this work, a Ge ion (LET = 37 MeV·cm^2^/mg) and Bi ion (LET = 98 MeV·cm^2^/mg) were used to irradiate Cascode GaN power devices by heavy ion accelerator experimental device. The differences of SEE under three conditions: pre-applied electrical stress, different LET values, and gate voltages are studied, and the related damage mechanism is discussed. The experimental results show that the pre-application of electrical stress before radiation leads to an electron de-trapping effect, generating defects within the GaN device. These defects will assist in charge collection so that the drain leakage current of the device will be enhanced. The higher the LET value, the more electron–hole pairs are ionized. Therefore, the charge collected by the drain increases, and the burn-out voltage advances. In the off state, the more negative the gate voltage, the higher the drain voltage of the GaN HEMT device, and the more serious the back-channel effect. This study provides an important theoretical basis for the reliability of GaN power devices in radiation environments.

## 1. Introduction

As the foundation and core of power electronic technology, power electronic devices play an important role in mobile communication, satellite positioning, electronic radar, national defense, and other aspects. The higher performance and reliability of electronic devices are directions of continuous research and pursuits of scientists. With the development of space technology, radiation and other extreme environments have established higher requirements for the reliability of power electronic devices. As the third generation of wide band gap semiconductor materials, GaN and SiC have broken through the limits of traditional silicon-based devices and have the characteristics of high breakdown voltage, low on-resistance, high temperature, and high voltage resistance, and they have become research hotspots in the field of power electronic devices. SiC has the characteristics of high-temperature resistance and low on-resistance, which dominate in high-power applications [1,2,3,4]. Compared with SiC, GaN devices have the advantages of lower switching loss, higher electron mobility, and faster switching speed, and they have good application prospects in high-frequency fields [5,6,7,8]. In the space environment, there are a large number of radiation sources, such as rays, protons, neutrons, and heavy ions [9]. The electronic devices on the space equipment will be affected by radiation, and the electrical performance will change instantaneously or even burn down, which seriously threatens the stability of the equipment [10,11,12,13]. Although GaN material itself has good radiation resistance, it will inevitably introduce defects in the device manufacturing process [14]. The radiation resistance of GaN devices is limited, which hinders the further application of GaN HEMT devices in the space environment.

In a complex radiation environment, the damage mechanism and degradation degree of the device caused by heavy ions are also different. This depends on many conditions, such as the mass and energies of the radiation particles, the position and angle of the particle incident device, and the bias state of the device during radiation. Bazzoli et al. used a heavy ion radiated GaN HEMT device with a low LET value and found that the device had soft damage and the gate current increased significantly under negative gate voltage [15]. Kuboyama et al. used three different heavy ion radiation AlGaN/GaN HEMT devices and found that the degradation trend of the devices was different. The leakage path between the drain and the source appears in the device under Kr ion irradiation [16]. Abbate conducted an SEE experiment on the enhanced GaN device. It was found that the drain leakage current of the device would increase permanently under the influence of a charge amplification mechanism [17]. Zeraka et al. used TCAD simulation software to study the influence of heavy ions incident at different positions (source, gate, between gate and drain, drain) and different LET values. They found that the leakage current is the largest when the incident position is at the gate edge, but the simulation data under different LET values were different from the actual results [18]. Fleetwood et al. found that the single event burning (SEB) phenomenon caused by heavy ion incident devices is related to the LET value, and the larger the LET value, the lower the threshold voltage when the SEB occurs [19]. Wang et al. studied the variation of the single event transient current of heavy ions under various conditions, such as different gate bias and different LET values by simulation, and analyzed the related physical mechanism [20].

At present, studies on GaN HEMT devices under different heavy ion radiation conditions have not produced systematic research results and mechanism explanations. In this paper, the SEE of GaN HEMT devices under three conditions, pre-applied electric stress, different heavy ion LET values, and different gate voltages, were studied through heavy ion experiments, and the simulation software was combined to analyze the experimental results. The experimental results provide a theoretical basis for the application of GaN HEMT devices in complex environments.

## 2. Experimental Condition

The SEE experiments were carried out in Harbin and Beijing heavy ion accelerator facilities. The radiated ions are Ge (Germanium) ions with an energy of 205 MeV and Bi (Bismuth) ions with an energy of 1100 MeV. The linear energy transfer (LET) values of Ge ions and Bi ions are 37 and 98 MeV·cm^2^/mg (in silicon), respectively. In the radiation experiment, the device in the off state has a drain bias of 25~200 V constant voltage, and the bias duration is about 100 s. The cumulative injection volume of each stage reaches 1 × 10^6^ ions/cm^2^. The source current I_s_, gate current I_g_, and drain current I_d_ of the radiation process the monitoring device.

**Figure 1:**
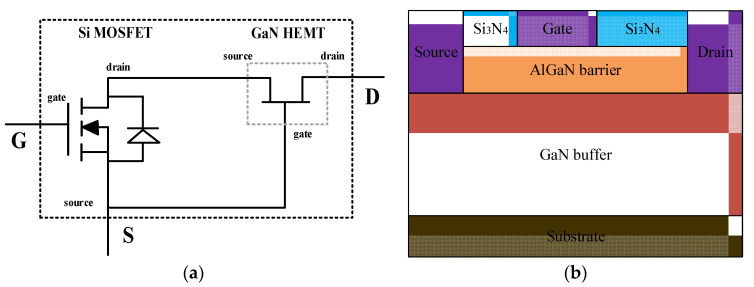
(**a**) Cascode GaN HEMT structure diagram; (**b**) depletion GaN HEMT structure diagram.

In this paper, the single event effect of Cascode GaN HEMT is studied, and the experimental results are verified by simulation. Figure 1a shows the structure of the Cascode device, which consists of a high-voltage depletion GaN HEMT and low-voltage enhanced Si MOSFET. Its advantages include the low switching loss of GaN and low gate charge of NMOS [21]. The gate and source of the Si MOSFET device and the drain of the GaN HEMT device are, respectively, used as the gate (G), source (S), and drain (D) of Cascode HEMT. Within the circuit structure, the source of the GaN HEMT device is connected to the drain of the Si MOSFET device, and the gate of the GaN HEMT device is connected to the source of the Si MOSFET device. Figure 1b shows the structure of a common depletion GaN HEMT in a Cascode GaN device.

The working principle of the Cascode circuit is (1) when Si MOSFET is in the on-state, the positive drain voltage of Si MOSFET is close to 0 V, and the negative gate voltage of GaN HEMT is close to 0 V and greater than the threshold voltage, the GaN HEMT is switched on. The drain current of the Cascode device can be obtained according to the formula $V_{ds}=I_{d}\left(R_{ds\_on(Si)}+R_{ds\_on(GaN)}\right)$. It can be seen that the drain current is related to the on-resistance and drain voltage. As the gate voltage of Si MOSFET continues to rise, the channel resistance *R_ds_on_*_(*Si*)_ continues to decrease, and the drain current of the Cascode GaN device also continues to rise. When the channel resistance is reduced to the saturation on-resistance, the drain current also reaches the maximum saturation current. (2) When the Si MOSFET is turned off and the drain voltage of the Cascode circuit is greater than the absolute value of the GaN device threshold voltage, the depletion GaN HEMT driving voltage is less than its threshold voltage. At this point, the depletion GaN HEMT is in the off state.

Since the single event effect in the off state is studied in this paper, the working principle of the off state is analyzed in depth. When the Cascode circuit is in the off state, both the MOSFET and GaN are in the off state. The drain voltage is shared by Si MOSFET and GaN, and their respective drain voltage is determined by their off-state impedance.

**Figure 2:**
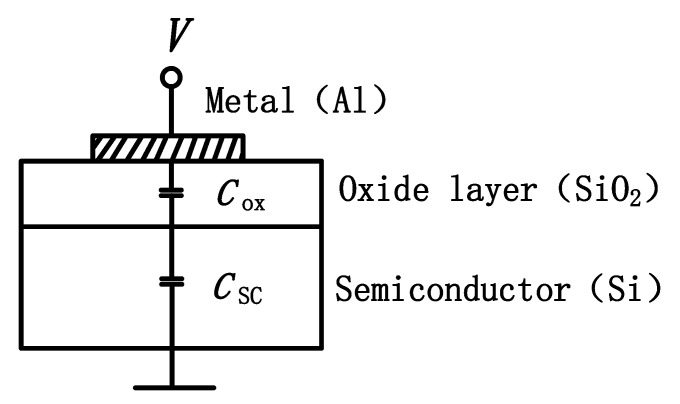
MOS structure.

When the gate voltage is applied to the MOS device, part of the gate voltage falls on the oxide layer, and the other part falls on the space charge layer on the semiconductor surface, forming the surface potential. When the gate voltage changes, the surface charge on the metal electrode and the space charge layer on the semiconductor surface will change, and this charge shows a certain capacitance effect with the change of voltage. It can be seen from Figure 2 that the MOS capacitor can be equivalent to the series combination of the oxide layer capacitor *C*_ox_ and the space charge layer capacitor *C*_sc_ on the semiconductor surface. The oxide layer capacitance *C*_ox_ is a constant capacitance independent of gate voltage, while the surface space charge layer capacitance *C*_sc_ is a function of gate voltage.

**Figure 3:**
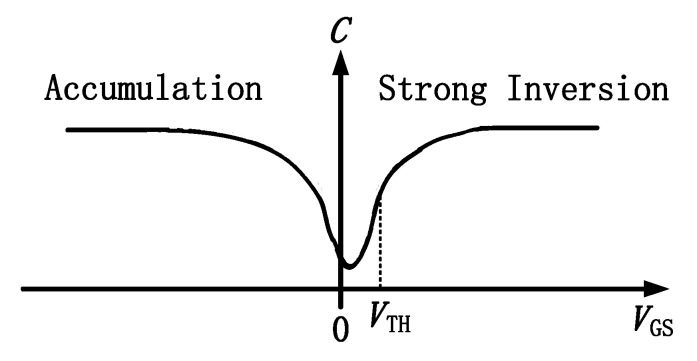
MOS capacitance–voltage characteristics.

Figure 3 shows the capacitance–voltage characteristics of MOS devices. As the absolute value of negative gate voltage applied by MOS devices increases, a large number of hole charges in the substrate are attracted to the interface of the oxide layer, and the holes accumulate on the surface of the oxide layer. As the capacitance *C*_sc_ of the space charge layer on the semiconductor surface increases, the MOS capacitance also increases.

The impedance of capacitance is calculated as $X_{c}=1/(2\pi fC)$, where *f* is the frequency, and *C* is the capacitance value. The more negative the gate voltage applied to the Si MOSFET device, the larger the capacitance value and the lower the off-state impedance. Therefore, the more negative the gate voltage applied to the Cascode circuit, the smaller the impedance of the Si MOSFET device, the smaller the drain voltage allocated to it, and the larger the drain voltage allocated to the GaN HEMT.

To sum up, when the Cascode circuit is switched on, the drain current increases as the gate voltage increases until the maximum saturation current is reached. This is consistent with the trend of drain current variation with gate voltage in common HEMT devices. When the Cascode circuit is turned off, both the MOSFET and GaN are turned off. As the gate voltage becomes more negative, the drain voltage allocated to GaN devices increases, and the electric field intensity between the gate and drain increases. The change of gate voltage of the Cascode device in the off state is equivalent to the change of drain voltage of GaN HEMT. Therefore, in this paper, when studying the influence of different gate voltages on the single event effect, different drain voltages of GaN HEMT devices are used for simulation.

In order to explain the phenomena observed in this study more accurately, some typical GaN power device physical models are used in the simulation. For GaN HEMT, which is based on the polarization effect, a polarization model is added to the simulation [22]. Considering the recombination, mobility change, and particle impact ionization in practical devices, the recombination model, mobility model, impact ionization model, and Fermi–Dirac carrier statistical model are introduced [23]. In addition, a heavy ion bombardment model is introduced for heavy ion incidence [24]. In the heavy ion incidence model, it is necessary to set the single entry point, exit point, ion radius, LET value, and other related parameters. The carrier distribution function generated by incident heavy ions is

(1) $$T\left(t\right)==\frac{2\times exp[-{\left(\frac{t-T_{0}}{T_{c}}\right)}^{2}]}{T_{c}\times \sqrt{\pi }[1+erf(\frac{T_{0}}{T_{c}})]}$$

where T(t) is the carrier distribution function, $T_{0}$ is the peak time of particle pulse generation, $T_{c}$ is the length of particle pulse generation, and erf is the error function.

## 3. Experimental Result

### 3.1. Influence of Different Stress Conditions before Radiation on SEE of Device

**Figure 4:**
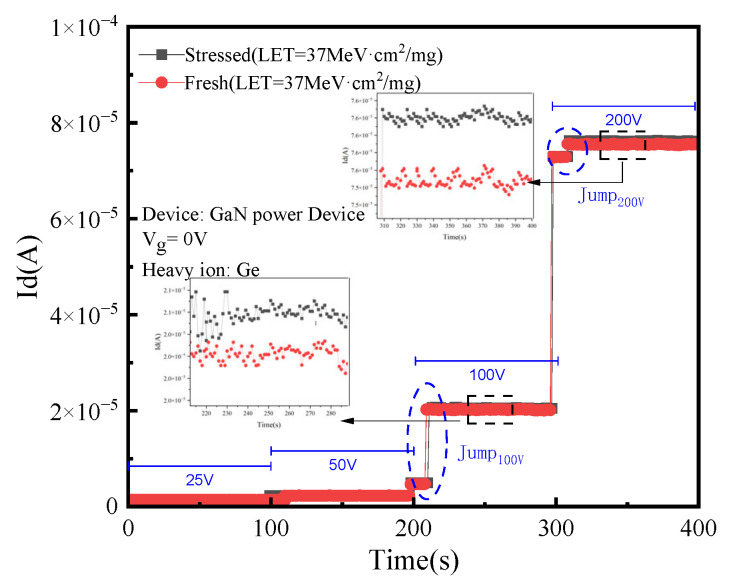
Results of SEE under different preprocessing conditions.

In order to explore the effect of drain electrical stress on the SEE of GaN HEMT devices, drain voltage with a size of 40 V and a duration of 2000 s was applied to the experimental devices in the off state before radiation and compared with the fresh devices. The radiation condition is Ge ions (LET = 37 MeV·cm^2^/mg). The bias condition and radiation injection amount are consistent during the experiment, and the radiation results are shown in Figure 4. The blue dash circles mark the current jump, and the black dash squares show the location of the partially enlarged image.

As can be seen from Figure 4, both groups of devices have irrecoverable current jumps when the drain voltage is 100 V. The current of the pre-applied electrical stress device is slightly higher than that of the fresh device when the drain voltage is 100 V and 200 V. This is because when there is a heavy ion incident in GaN HEMT devices, a large number of electron–hole pairs will be generated in the incident track of ions. For pre-stressed devices, acceptor defects caused by electrical stress provide leakage channels for electrons and play an auxiliary role in charge leakage. The leakage behavior of the device increases, and the charge collected by the drain increases.

**Figure 5:**
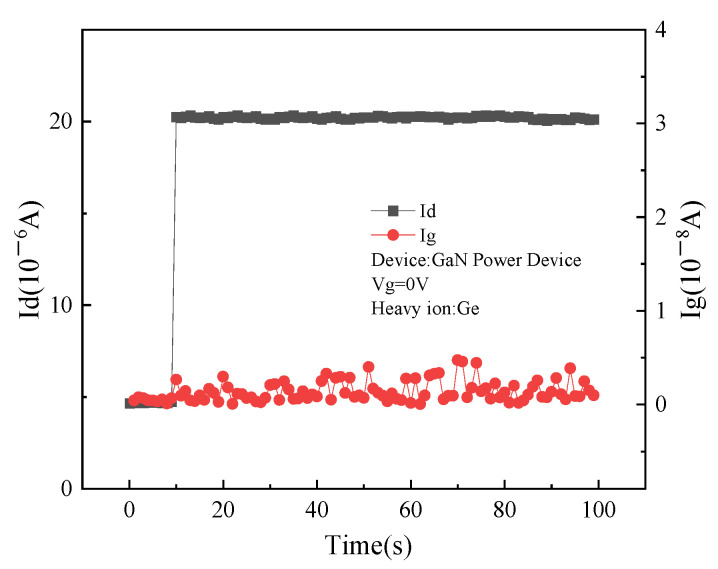
Changes of gate and drain current with time when drain voltage is 100 V.

When the gate voltage of the device is 0 V and the drain voltage is 100 V, the change of drain current I_d_ and gate current I_g_ with the radiation time is shown in Figure 5. When heavy ions incident into the device, the drain current appears as a transient jump and does not recover with the increase in radiation time. The gate current basically remains unchanged, always at the level of nanoampere. This means that in the off state, during the incident of heavy ions, the gate and source of the device maintain a cut-off state, while a leakage path appears between the drain and source. Based on the analysis of the circuit structure of Cascode GaN HEMT in Figure 1, when the device is in the off state, the low-voltage-enhanced Si MOSFET device has a reverse bias cut-off. At this time, a leakage path is formed at the drain and gate of the depletion GaN HEMT and the source of the Si MOSFET.

**Figure 6:**
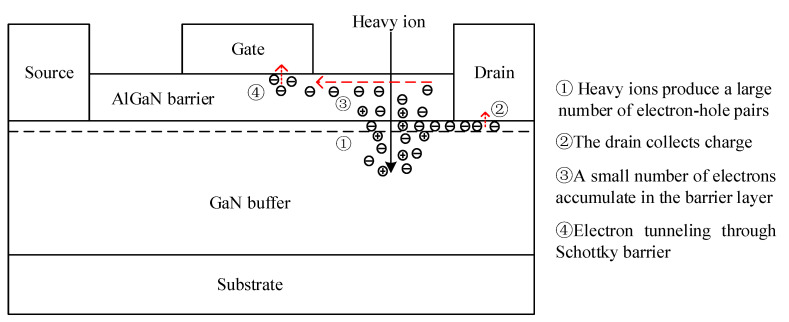
Schematic diagram of charge flow inside the device after incident heavy ions.

As shown in Figure 6, when there is a heavy ion incident in GaN HEMT, a large number of electron–hole pairs are bound to be generated on the particle incident track. Most of the charges are collected by the drain, and a small part of the charges gather on the upper surface of the AlGaN barrier layer under the action of the device’s high gate-drain field. With the constant accumulation of electrons in the barrier layer, the width of the Schottky barrier formed by the gate electrode and the semiconductor decreases, which causes the electrons to tunnel through the gate barrier. Therefore, the current leakage path from the drain and gate of the depletion GaN HEMT to the source of the Si MOSFET is formed [25]. The red dash arrows represent the direction of charge flow.

**Figure 7:**
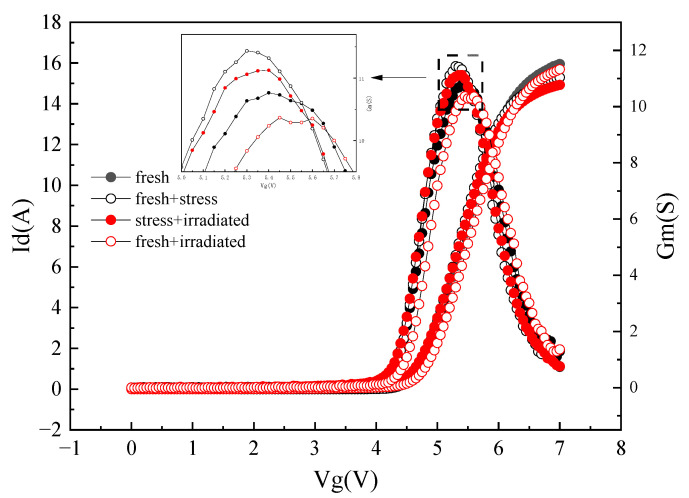
The transfer curve and transconductance curve of the device before and after radiation under different preprocessing conditions.

Figure 7 shows the transfer and transconductance curves of the device before and after radiation under different pretreatment conditions. It can be seen that the threshold voltage of the device basically does not change, which is consistent with the above SEE experiment analysis results; that is, the gate of the device is basically not damaged.

The phenomenon of a decrease in maximum transconductance occurred after heavy ion radiation. This is because the heavy ion incident device will collide with AlGaN or GaN layer materials, resulting in a large number of lattice damage defects. During device conduction, these traps will trap electrons in the channel and act as scattering centers, reducing the 2DEG mobility of the device and decreasing the maximum transconductance.

It can be seen in Figure 7 that the maximum transconductance increases after the drain electrical stress is applied. By analyzing the characteristic degradation trend of the device, it is found that this is different from the characteristic degradation trend of GaN HEMT devices caused by the converse piezoelectric effect in the off state. Relevant studies have found that the converse piezoelectric effect will occur only when the bias voltage applied by the device in the off state reaches a certain threshold [26].

**Figure 8:**
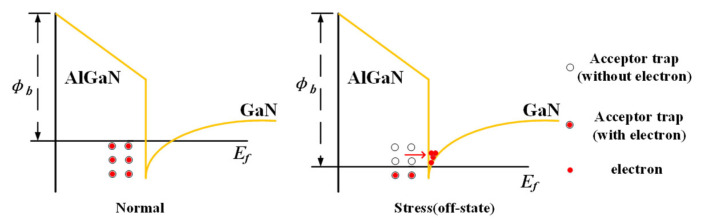
Schematic diagram of electron de-trapping effect.

Due to the small drain stress applied in the off state in this experiment, the threshold of the converse piezoelectric effect was not reached. Combined with the experimental results, the electron re-trapping effect occurred in the experimental device, and the degradation mechanism is shown in Figure 8. The red dash arrows represent the direction in which the electrons are moving. In the AlGaN barrier layer, the acceptor trap located below the Fermi level is filled with electrons. When the off-state electrical stress is applied to the device, the energy band at AlGaN/GaN heterojunction rises, and part of the accepter trap is above the Fermi level. At this time, the electrons exit the trap and enter the channel. The acceptor trap without the electron is electrically neutral and cannot play the role of scattering center. Therefore, the 2DEG mobility increases, and the maximum transconductance of the device increases.

For the pre-stressed device, the ionized electron–hole pair will be trapped by the acceptor trap generated by the electrical stress. The acceptor trap trapping electrons in the channel will play the role of the scattering center again, which reduces the 2DEG in the channel and the maximum transconductance of the device. With the increase in heavy ion radiation dose, the transconductance will further decrease. This can even exceed transconductance for fresh devices.

### 3.2. Influence of Different LET Values on SEE of Devices

**Figure 9:**
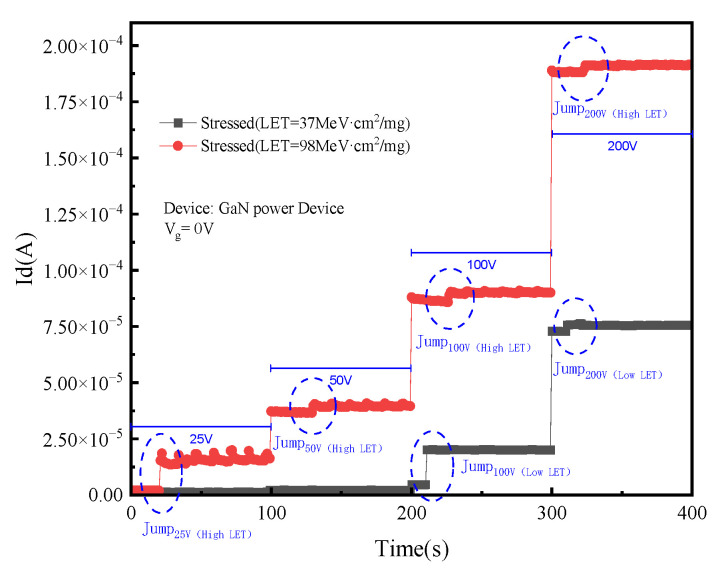
Influence of heavy ions with different LET values on SEE.

When there is a high-energy particle incident in GaN HEMT devices, they will interact with atoms inside the devices and lose some energy. This energy loss is called the linear energy transfer value (LET). After the atoms inside the device materials obtain the transferred energy, the electronic transition will occur, and electron–hole pairs will be generated on the track of the incident particles. The influence of heavy ions with different LET values on the SEE is also different. Figure 9 shows how the drain current varies with time under the radiation of Ge ions (LET = 37 MeV·cm^2^/mg) and Bi ions (LET = 98 MeV·cm^2^/mg) when the gate voltage is 0 V.

Under lower LET heavy ion irradiation, the drain current increases from 5 × 10^6^ A to 2.1 × 10^5^ A when the drain voltage is 100 V. When the LET value rises to 98 MeV·cm^2^/mg, the current jump occurs when the drain voltage is 25 V, and the drain current increases from 2 × 10^6^ A to 1.4 × 10^5^ A. Although the two experimental devices and bias conditions are the same, the incident particle LET value has a significant influence on the SEE of the device during the experiment. Under the same drain voltage, the drain current with a lower LET value is significantly lower than that with a higher LET value.

**Figure 10:**
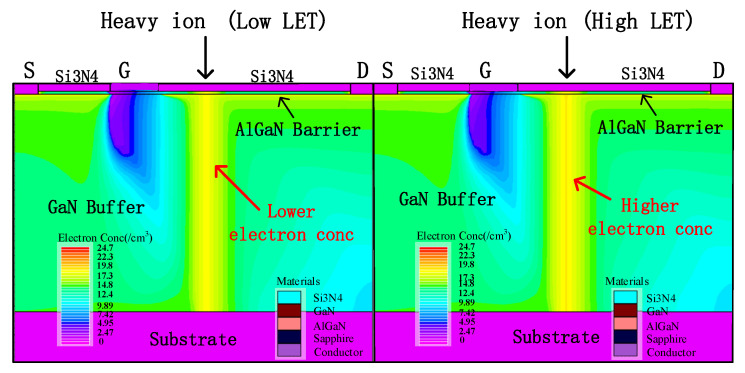
The electron concentration distribution inside the device at 1 ps when heavy ions cause an incident with different LET values.

**Figure 11:**
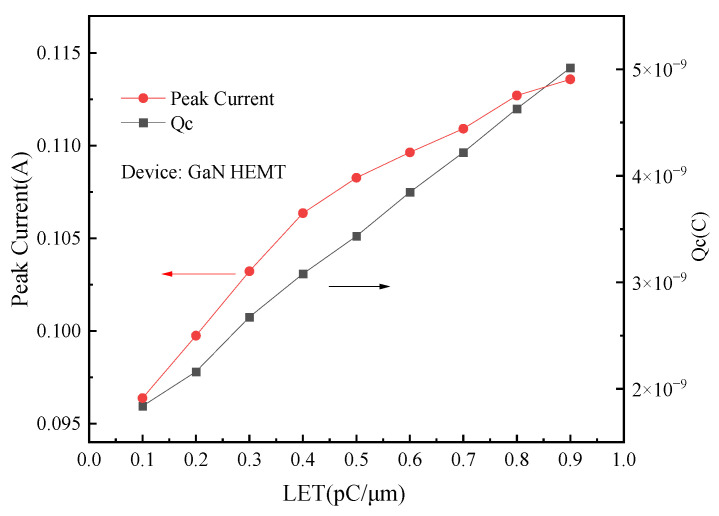
Single event transient current peak and charge collection distribution of heavy ions incident with different LET values.

Figure 10 shows the simulation results of electron distribution inside the device when particles with different LET values incident at 1 ps. When the LET value of the incident particles increases, more electron–hole pairs will be generated in the incident track inside the device. Figure 11 simulates the peak value of a single-event transient current and the collected charge generated when ions with different LET values cause an incident in a device. With the increase in LET value, the amount of charge that can be collected by the drain increases at the same time, and the pulse current detected by the drain also increases. For a fixed GaN HEMT device, the mean ionization energy of the material is close to constant. The number of electron–hole pairs generated by a fixed LET value of heavy ions is constant. Due to the high voltage bias applied by the drain, the recombination probability of the carrier generated by radiation is small, and most of the charge is collected by the drain to form a transient current. Therefore, the peak value of the single event transient current and the collected charge of the drain show an approximate linear increase trend with the LET value.

### 3.3. Influence of Different Gate Voltages on SEE of Devices

**Figure 12:**
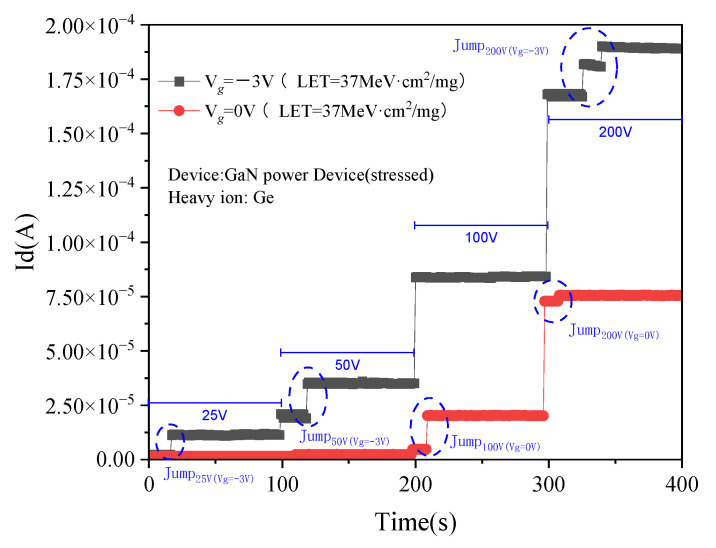
SEE of device under different gate voltage bias.

Previous studies have found that when GaN HEMT devices are in the on state, the electron–hole pairs generated by incident-heavy ions are collected through the channel. The current of the source, gate, and drain does not surge, and the electrical performance of the device is not damaged. When the device is in the off state, the 2DEG in the channel is exhausted, and the electron–hole pairs ionized by the heavy ion radiation cannot be collected through the channel. When the charge accumulates to a certain extent, a leakage path will be introduced in the GaN layer. The transient current will surge, and the device characteristics will be damaged or even burned. As a result, the device is most seriously damaged under the off-state [27]. In this research, the gate voltages are set to 0 V and −3 V, respectively, during the radiation period, and the influence of different gate voltages on the SEE is observed. Ge ions (LET = 37 MeV·cm^2^/mg) were used in the experiment, and the experimental results are shown in Figure 12.

As can be seen from Figure 12, the gate voltage has a greater impact on the SEE. Devices with a gate voltage of −3 V require a lower drain voltage to make a current jump, which occurs at a drain voltage of 25 V. When the same drain voltage is applied, the more negative the gate voltage, the greater the drain current.

Just as in the previous analysis of the working principle in the off state, changing the gate voltage of the Cascode is equivalent to changing the drain voltage of GaN HEMT devices. In order to explore the influence mechanism of different negative gate voltages on the device, the single event transient effects of GaN devices under different drain voltages are simulated below.

**Figure 13:**
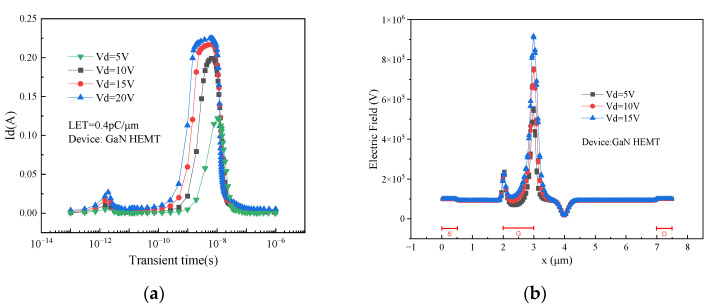
(**a**) Single event transient effects of devices with different drain voltage; (**b**) electric field distribution under different drain voltage.

Figure 13a shows the simulation results of the single event transient effects of GaN HEMT devices at different drain voltages. With the increase in drain voltage, the single event transient current increases, and the peak of transient current advances. As shown in Figure 13b, the increase in drain voltage leads to the enhancement of the electric field in the gate region, and the electron and hole drift faster. The holes are more likely to accumulate under the gate, which strengthens the back-channel effect, and the charge enhancement is advanced.

**Figure 14:**
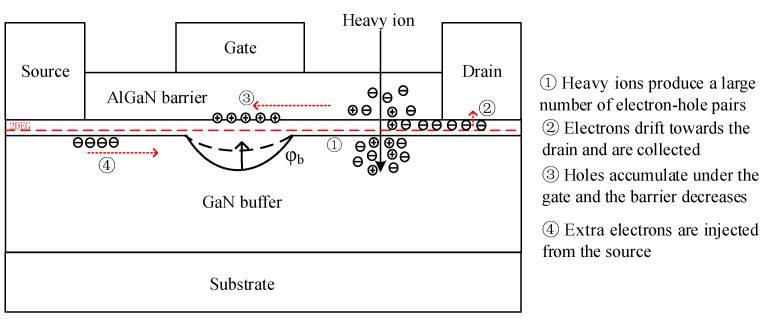
Diagram of back-channel effect.

Figure 14 shows the diagram of the back-channel effect. The red arrow shows the direction of charge flow. A large number of electron–hole pairs are generated after a particle incident on the device. Due to the action of the drain-source electric field, electrons and holes drift. Among them, the mobility of electrons is relatively high, which can move quickly to the drain and be collected by the drain to generate transient currents. However, the mobility of the holes is low, and they can only move slowly toward the gate direction, and a higher concentration of holes accumulate under the gate. The accumulation of a large number of holes reduces the barrier between the source and the region below the gate, allowing extra electrons to flow from the source into the device and be collected by the drain.

## 4. Conclusions

In this paper, the SEE of Cascode-enhanced GaN HEMT devices under different conditions were studied by using heavy ion accelerator experimental equipment. The damage mechanism of SEE experimental results was discussed by means of simulation software. The experimental results show that the pre-application of drain electrical stress will produce defects inside the GaN device. These defects will assist the leakage of charge and increase the leakage current when the device is irradiated by heavy ions. The LET value of heavy ions will affect the number of electron–hole pairs ionized in the device. The higher the LET value, the larger the leakage current will be detected, and the device will have a current jump at a smaller voltage. The more negative the gate voltage applied by the device during heavy ion radiation, the larger the MOSFET capacitance and the larger the drain voltage of GaN HEMT. High drain voltage will make the back-channel effect worse.

## Data Availability

The data presented in this study are available on request from the corresponding author.
